# Supplementary material for: Sex-dependent effects of Setd1a haploinsufficiency on development and adult behaviour
Source: PLoS One. 2024 Aug 14;19(8):e0298717. doi: 10.1371/journal.pone.0298717 (PMC11324134; doi:10.1371/journal.pone.0298717)
Supplement: S1 Fig — (DOCX) [file pone.0298717.s001.docx]

**Sex-dependent effects of *Setd1a* haploinsufficiency on development and adult behaviour**

Matthew L. Bosworth^1^, Anthony R. Isles^1^, Lawrence S. Wilkinson^1,2,3^, & Trevor Humby^1,2,3^*

^1^MRC Centre for Neuropsychiatric Genetics and Genomics, Division of Psychological Medicine and Clinical Neuroscience, School of Medicine, Cardiff University, Cardiff, UK

^2^School of Psychology, Cardiff University, Cardiff, UK

^3^Neuroscience and Mental Health Research Institute, Cardiff University, Cardiff UK

*Corresponding author: Dr Trevor Humby [HumbyT@cardiff.ac.uk](mailto:HumbyT@cardiff.ac.uk) Tel. +44(0)2920 876758

**S1 Fig: Confirmation of *Setd1a* haploinsufficiency in the *Setd1a*^+/-^ model.**

| 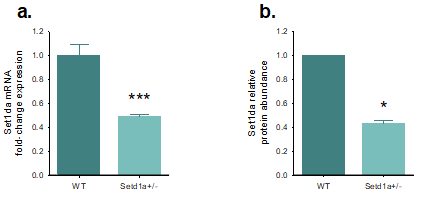 |
| --- |
| To confirm *Setd1a* haploinsufficiency in our model, levels of *Setd1a* mRNA and protein were quantified in whole brains dissected at E13.5 N=8 for WT and *Setd1a*^+/-^ tissue). Haploinsufficiency was confirmed in the *Setd1a^+/-^* model, by demonstrating that *Setd1a* mRNA expression was reduced by 48.8% at E13.5 in *Setd1a^+/-^* brain compared to WT (Fig. 1c, t_14_=9.18, p<0.001), with comparable reductions in levels of SETD1A protein (46.3 % reduction) (Fig. 1d, t_8_=2.71, p=0.03). This magnitude of reduction is consistent with and confirmed that *Setd1a* knockdown to half of WT levels was successfully achieved. * and *** shows significant comparison to WT at p<0.05 and p<0.001, respectively. Data shows mean±SEM.  Methods: RNA extraction was performed using a Direct-zol^TM^ RNA Miniprep Kit (Zymo, UK). 1 μg total RNA was used for cDNA synthesis using RNA to cDNA EcoDry^TM^ Premix (double-primed) kits (Clontech, UK). qRT-PCR reactions were performed in triplicate using a Corbett Rotorgene 6000 Real-Time PCR machine with Sensimix SYBR No-Rox (Bioline, UK) and intron-spanning primers (see table).  The geometric mean of Ct values across three housekeeping genes (*Hprt*, *Dynein*, and *B2m*) were used as endogenous controls to normalise *Setd1a* expression levels using the ΔΔCT method^25^. Protein was extracted from brain homogenates in RIPA buffer (Sigma, UK) containing cOmplete^TM^ Mini Protease Inhibitor Cocktail (Roche, Switzerland). A Pierce^TM^ BCA Protein Assay kit (Thermo Scientific, UK) was used to quantify protein concentration. Samples were diluted in protein loading buffer (LI-COR, UK) containing 0.05 % (v/v) 2-Mercaptoethanol (Sigma, UK) and denatured by heating at 95 °C for five minutes. 20 μg total protein per sample was separated by SDS-PAGE using a NuPAGE^TM^ 4-12 % Tris-Acetate gel (Invitrogen, UK) and NuPAGE^TM^ Tris-Acetate SDS Running Buffer (Invitrogen, UK). Proteins were transferred to a 0.45 μm pore size nitrocellulose membrane (Invitrogen, UK) in NuPAGE^TM^ Transfer Buffer (Invitrogen, UK) containing 10 % (v/v) methanol (Fisher Scientific, UK). To enable normalisation of *SETD1A* protein abundance, membranes were stained for total protein using REVERT^TM^ Total Protein Stain (LI-COR, UK). Odyssey TBS Blocking Buffer (LI-COR, UK) was used to block membranes for one hour at room temperature. Membranes were incubated overnight at 4 °C with 1:1,000 polyclonal *Setd1a* antibody (Bethyl Laboratories, USA). TBS-T (1M NaCl, 1M Tris-HCl, 0.2 % (v/v) Tween 20) was used to wash the membrane four times (5 minutes per wash) before incubation in 1:10,000 IRDye 800CW goat anti-rabbit secondary antibody (LI-COR, UK) for one hour at room temperature. Wash steps were repeated prior to imaging using an Odyssey CLx and protein quantification using Image Studio software (LI-COR, UK). |


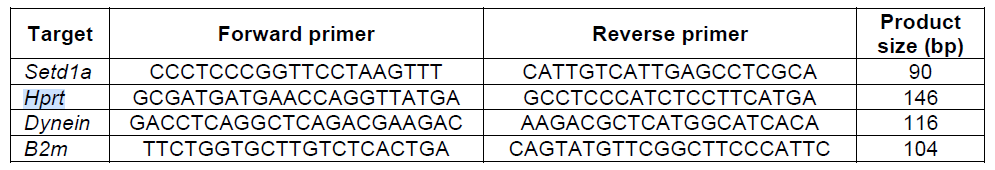


**End of document**
