# Supplementary material for: Sex-dependent effects of Setd1a haploinsufficiency on development and adult behaviour
Source: PLoS One. 2024 Aug 14;19(8):e0298717. doi: 10.1371/journal.pone.0298717 (PMC11324134; doi:10.1371/journal.pone.0298717)
Supplement: S2 Fig — (DOCX) [file pone.0298717.s002.docx]

**Sex-dependent effects of *Setd1a* haploinsufficiency on development and adult behaviour**

Matthew L. Bosworth^1^, Anthony R. Isles^1^, Lawrence S. Wilkinson^1,2,3^, & Trevor Humby^1,2,3^*

^1^MRC Centre for Neuropsychiatric Genetics and Genomics, Division of Psychological Medicine and Clinical Neuroscience, School of Medicine, Cardiff University, Cardiff, UK

^2^School of Psychology, Cardiff University, Cardiff, UK

^3^Neuroscience and Mental Health Research Institute, Cardiff University, Cardiff UK

*Corresponding author: Dr Trevor Humby [HumbyT@cardiff.ac.uk](mailto:HumbyT@cardiff.ac.uk) Tel. +44(0)2920 876758

**S2 Fig: Methods for RNAseq**

| RNA purity was assessed using a NanoDrop 8000 spectrophotometer (Thermo Fisher Scientific, UK). RNA concentration was quantified using a QubitTM RNA High Sensitivity Assay Kit and Qubit 2.0 Fluorometer (Invitrogen, UK). RNA integrity was assessed using a Bioanalyzer RNA 6000 Nano Assay (Agilent, UK) and 2100 Bioanalyzer system (Agilent, UK). All samples had an RNA Integrity Number of at least 9.7. Library preparations were performed using a KAPA mRNA Hyperprep kit (Roche, Switzerland) according to manufacturer’s instructions, with 1 μg total RNA as input. Following library amplification, fragment size (mean = 374.9, SD = 12.5) was determined using a High Sensitivity DNA kit (Agilent, UK). A QubitTM dsDNA High Sensitivity Assay Kit (Invitrogen, UK) was used to determine library concentration. Reads were trimmed to remove adapters and low-quality bases using Trimmomatic^1^ with default parameters. Reads were mapped to the mouse reference genome (GRCm38) using STAR^2^. The mean number of reads mapped was 97.2% (SD = 0.5%). Read counts were generated using featureCounts^3^ to allocate reads to genomic features using the mouse Ensembl gene annotation (GRCm.38.95). Coverage at exon 4 of *Setd1a* was substantially reduced in *Setd1a*+/- E13.5 brain, indicating that recombination had occurred (see figure, screenshot from Integrative Genomics Viewer showing substantially more reads aligning to exon 4 in WT (top) compared to *Setd1a*^+/-^ (bottom) mice). See main text for other details. |
| --- |
|  |
|  |
| **References**  1. Bolger, A. M., Lohse, M., & Usadel, B. (2014). Trimmomatic: a flexible trimmer for Illumina sequence data. *Bioinformatics*, *30*(15), 2114–2120. doi: 10.1093/bioinformatics/btu170  2. Dobin, A., Davis, C. A., Schlesinger, F., Drenkow, J., Zaleski, C., Jha, S., … Gingeras, T. R. (2013). STAR: ultrafast universal RNA-seq aligner. *Bioinformatics*, *29*(1), 15–21. doi: 10.1093/bioinformatics/bts635  3. Liao, Y., Smyth, G. K., & Shi, W. (2014). featureCounts: an efficient general purpose program for assigning sequence reads to genomic features. *Bioinformatics*, *30*(7), 923–930. doi: 10.1093/bioinformatics/btt656 |

**End of document**
