# Supplementary material for: Sex-dependent effects of Setd1a haploinsufficiency on development and adult behaviour
Source: PLoS One. 2024 Aug 14;19(8):e0298717. doi: 10.1371/journal.pone.0298717 (PMC11324134; doi:10.1371/journal.pone.0298717)
Supplement: S3 Fig — (DOCX) [file pone.0298717.s003.docx]

**Sex-dependent effects of *Setd1a* haploinsufficiency on development and adult behaviour**

Matthew L. Bosworth^1^, Anthony R. Isles^1^, Lawrence S. Wilkinson^1,2,3^, & Trevor Humby^1,2,3^*

^1^MRC Centre for Neuropsychiatric Genetics and Genomics, Division of Psychological Medicine and Clinical Neuroscience, School of Medicine, Cardiff University, Cardiff, UK

^2^School of Psychology, Cardiff University, Cardiff, UK

^3^Neuroscience and Mental Health Research Institute, Cardiff University, Cardiff UK

*Corresponding author: Dr Trevor Humby [HumbyT@cardiff.ac.uk](mailto:HumbyT@cardiff.ac.uk) Tel. +44(0)2920 876758

**S3 Fig: Replication of acoustic startle and prepulse inhibition effects (Main text Fig. 3a and 3b) in a separate cohort of *Setd1a*^+/-^ and WT mice.**

| 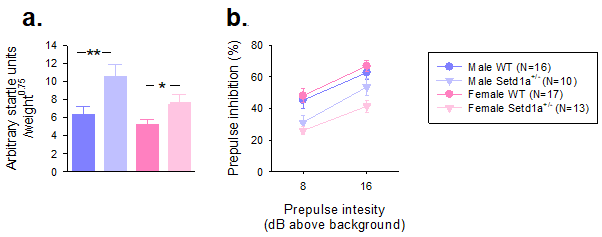 |
| --- |
| Consistent with the previous results (Main text Fig. 3a and 3b), there was a significant main effect of GENOTYPE on startle responding (a., F_1,52_=15.58, p<0.001) which further confirmed that startle magnitude was significantly greater in *Setd1a*^+/-^ mice than their WT littermates. The difference between male WT and *Setd1a*^+/-^ mice was greater than before (t_24_=2.89, p=0.008), and we also also observed a significant tendency for female *Setd1a*^+/-^ mice to have elevated startled responses in this replication study (t_28_=2.61, p=0.014). There was no GENOTYPE*SEX interaction (F_1,52_=1.01, p=0.32), as before. For prepulse inhibition, there was a significant main effect of GENOTYPE (F_1,52_=19.79, p<0.001) replicating the reduced PPI shown by *Setd1a*^+/-^ mice (b). The interaction between GENOTYPE*PPI-INTENSITY was not significant (F_1,52_=0.45, p=0.83) showing that this attenuation in PPI was consistent at both prepulse intensities used, even though the 16 dB prepulse induced greater PPI (main effect of PPI-INTENSITY, F_1,52_=94.76, p<0.001). Thus, these data replicate the initial findings and demonstrate that increased ASR (particularly in males) and decreased PPI is a robust phenotype of *Setd1a* haplosufficiency. * and ** shows significant main effect of GENOTYPE at p<0.05 and p<0.01, respectively. Data shows mean±SEM. |

**End of document**
