# Supplementary material for: Sex-dependent effects of Setd1a haploinsufficiency on development and adult behaviour
Source: PLoS One. 2024 Aug 14;19(8):e0298717. doi: 10.1371/journal.pone.0298717 (PMC11324134; doi:10.1371/journal.pone.0298717)
Supplement: S4 Fig — (DOCX) [file pone.0298717.s004.docx]

**Sex-dependent effects of *Setd1a* haploinsufficiency on development and adult behaviour**

Matthew L. Bosworth^1^, Anthony R. Isles^1^, Lawrence S. Wilkinson^1,2,3^, & Trevor Humby^1,2,3^*

^1^MRC Centre for Neuropsychiatric Genetics and Genomics, Division of Psychological Medicine and Clinical Neuroscience, School of Medicine, Cardiff University, Cardiff, UK

^2^School of Psychology, Cardiff University, Cardiff, UK

^3^Neuroscience and Mental Health Research Institute, Cardiff University, Cardiff UK

*Corresponding author: Dr Trevor Humby [HumbyT@cardiff.ac.uk](mailto:HumbyT@cardiff.ac.uk) Tel. +44(0)2920 876758

**S4 Fig: Pilot studies for pharmacological investigations**

|  |
| --- |
| 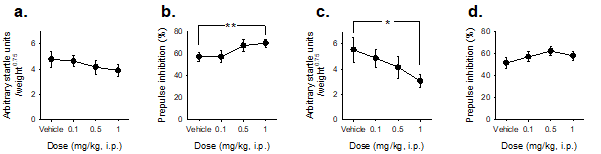  Previous work in rodent models has shown that PPI deficits induced by D2 receptor agonists are reversed by both haloperidol and risperidone, whereas NMDAR antagonist induced deficits are generally insensitive to haloperidol but can be reversed by risperidone^1,2^, therefore we used these drugs to gauge their effects on *Setd1a*^+/-^ mice. Using dose ranges, and a 30 min pre-test delay, from this literature with an aim to test effects of haloperidol and risperidone in WT mice to identify an appropriate dose range for the main experiment. Two groups of 3 months old male C57BL/6J mice (N=17 and N=16, for the haloperidol and risperidone studies, respectively) were used, using a protocol consisting of 25 trials, with 120 dB pulse-alone startle stimuli and 8 and 16dB prepulse trials (see main text for further description) (for analysis, PPI was pooled across 8 and 16 dB above background prepulses). Haloperidol did not significantly affect acoustic startle responding (a, main effect of DOSE, F_3,48_=1.43, p=0.25), however the 1.0mg/kg dose significantly increased PPI relative to vehicle (b, main effect of DOSE, F_3,48_=4.11, p=0.01), confirmed by *post hoc* testing (p=0.04 for the comparison between vehicle and 1.0mg/kg dose, other comparisons were n.s.). Administration of risperidone significantly reduced the startle response (c, main effect of DOSE, F_3,45_=6.42, p=0.001). *Post-hoc* tests revealed a significantly reduced startle response at 1.0 mg/kg (p=0.03) but not with the other doses of risperidone. Risperidone did not affect PPI at any dose (main effect of DOSE, F_3,45_=2.12, p=0.15). These results showed that 0.5 mg/kg was the highest dose of haloperidol and risperidone tested that did not have significant effects on basal sensorimotor gating in WT mice. Thus, in the main experiment, all mice were tested at this dosage in the first instance to explore drug effects on sensorimotor gating in *Setd1a*+/- mice in the absence of non-specific effects. * and ** shows significant main effect of DOSE at p<0.05 and p<0.01, respectively Data shows mean±SEM  1. Geyer, M. A., Krebs-Thomson, K., Braff, D. L., & Swerdlow, N. R. (2001). Pharmacological studies of prepulse inhibition models of sensorimotor gating deficits in schizophrenia: a decade in review. *Psychopharmacology*, *156*(2–3), 117–154. doi: 10.1007/s002130100811  2. Swerdlow, N. R., Talledo, J., Sutherland, A. N., Nagy, D., & Shoemaker, J. M. (2006). Antipsychotic Effects on Prepulse Inhibition in Normal ‘Low Gating’ Humans and Rats. *Neuropsychopharmacology*, *31*(9), 2011–2021. doi: 10.1038/sj.npp.1301043 |

**End of document**
