# Supplementary material for: Sex-dependent effects of Setd1a haploinsufficiency on development and adult behaviour
Source: PLoS One. 2024 Aug 14;19(8):e0298717. doi: 10.1371/journal.pone.0298717 (PMC11324134; doi:10.1371/journal.pone.0298717)
Supplement: S5 Fig — (DOCX) [file pone.0298717.s005.docx]

**Sex-dependent effects of *Setd1a* haploinsufficiency on development and adult behaviour**

Matthew L. Bosworth^1^, Anthony R. Isles^1^, Lawrence S. Wilkinson^1,2,3^, & Trevor Humby^1,2,3^*

^1^MRC Centre for Neuropsychiatric Genetics and Genomics, Division of Psychological Medicine and Clinical Neuroscience, School of Medicine, Cardiff University, Cardiff, UK

^2^School of Psychology, Cardiff University, Cardiff, UK

^3^Neuroscience and Mental Health Research Institute, Cardiff University, Cardiff UK

*Corresponding author: Dr Trevor Humby [HumbyT@cardiff.ac.uk](mailto:HumbyT@cardiff.ac.uk) Tel. +44(0)2920 876758

**S5 Fig: Trajectory of Setd1a expression across neurodevelopment in WT mice.**

|  |
| --- |
| 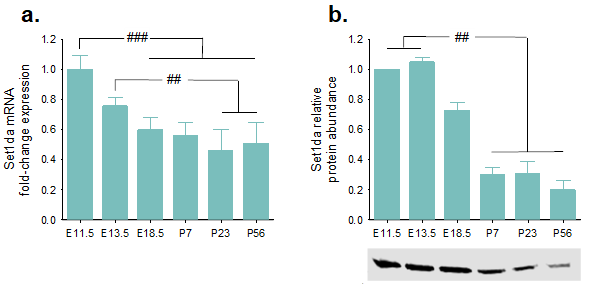  To determine the trajectory of Setd1a expression across neurodevelopment whole brains (E11.5, E13.5 and E18.5) or hemibrains (P7, P23 and P56), from at least two separate litters per timepoint, were obtained. Timed-matings were conducted between C57BL/6J x C57BL/6NTac parents and pregnant dams were culled by cervical dislocation. Sample analysis followed the methods detailed in Supplementary Methods Figure 1. Setd1a expression significantly reduced with age (a, main effect of AGE, F_5,30_=11.65, p<0.001) in the brains of WT mice. *Post-hoc* tests showed that Setd1a expression levels were significantly higher at E11.5 than other timepoints (all p<0.002), except E13.5 (p=0.26). Expression levels at E13.5 were not significantly different from E18.5 (p=0.32) or P7 (p=0.08) but were significantly higher than at P23 (p=0.003) and P56 (p=0.02). There was no further significant decline in Setd1a expression levels after E18.5 (all p>0.33). Similar results were obtained at the protein level (b) with a significant main effect of AGE (F_5,24_=6.78, p<0.001), which reflected significantly increased Setd1a expression at E11.5 and E13.5 relative to postnatal timepoints (all p<0.02). No significant difference was observed between E11.5 and E13.5 or E18.5 (both p>0.60). Protein levels did not change after E18.5 (all p>0.17). Together, these data suggest that Setd1a expression levels are higher in the developing brain during (mid) gestation, followed by down-regulation in the postnatal brain. ## and ###, signify significant main effects of AGE at p<0.01 and P<0.001, respectively. Data shows mean±SEM. |

**End of document**
