# Supplementary material for: Sex-dependent effects of Setd1a haploinsufficiency on development and adult behaviour
Source: PLoS One. 2024 Aug 14;19(8):e0298717. doi: 10.1371/journal.pone.0298717 (PMC11324134; doi:10.1371/journal.pone.0298717)
Supplement: S6 Fig — (DOCX) [file pone.0298717.s006.docx]

**Sex-dependent effects of *Setd1a* haploinsufficiency on development and adult behaviour**

Matthew L. Bosworth^1^, Anthony R. Isles^1^, Lawrence S. Wilkinson^1,2,3^, & Trevor Humby^1,2,3^*

^1^MRC Centre for Neuropsychiatric Genetics and Genomics, Division of Psychological Medicine and Clinical Neuroscience, School of Medicine, Cardiff University, Cardiff, UK

^2^School of Psychology, Cardiff University, Cardiff, UK

^3^Neuroscience and Mental Health Research Institute, Cardiff University, Cardiff UK

*Corresponding author: Dr Trevor Humby [HumbyT@cardiff.ac.uk](mailto:HumbyT@cardiff.ac.uk) Tel. +44(0)2920 876758

**S6 Fig: Acoustic startle response analysis per trial.**

| 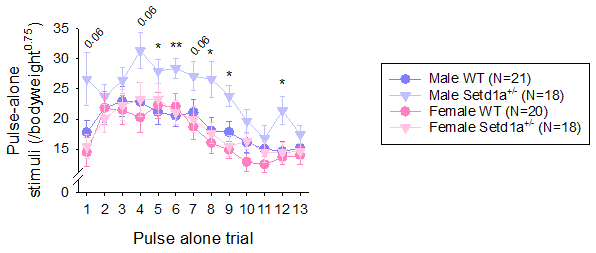 |
| --- |
| Analysis of the individual pulse-alone trials demonstrate that male Setd1a^+/-^ mice showed elevated startle responding across the majority of trials as represented by significant main effects of GENOTYPE (F_1,72_=4.51, p=0.04) and SEX (F_1,72_=6.29, p=0.01). Simple effects analysis shows that in the male data the effect of GENOTYPE was significant (F_1,37_=6.29, p=0.02), whereas there were no differences between female WT and Setd1a^+/-^ mice (F_1,35_=0.18, p=0.67) and all individual comparisons were non-significant (p>0.05). *post hoc* comparisons between male WT and Setd1a^+/-^ mice, indicated on the graph, show that male Setd1a^+/-^ mice show an increased response to the first startle stimulus, and then hyperstartle and demonstrate reduced habituation relative to male WT, and female mice through the remainder of the session. Note: mean of trials 4 to 13 is presented in the main paper figure 3a. * and **, signify significant main effects of GENOTYPE at p<0.05 and P<0.01 for comparison of male mice, respectively. Data shows mean±SEM. |

**End of document**
