# Supplementary material for: Sex-dependent effects of Setd1a haploinsufficiency on development and adult behaviour
Source: PLoS One. 2024 Aug 14;19(8):e0298717. doi: 10.1371/journal.pone.0298717 (PMC11324134; doi:10.1371/journal.pone.0298717)
Supplement: S7 Fig — (DOCX) [file pone.0298717.s007.docx]

**Sex-dependent effects of *Setd1a* haploinsufficiency on development and adult behaviour.** Matthew L. Bosworth^1^, Anthony R. Isles^1^, Lawrence S. Wilkinson^1,2,3^, & Trevor Humby^1,2,3^*

*Corresponding author: Dr Trevor Humby [HumbyT@cardiff.ac.uk](mailto:HumbyT@cardiff.ac.uk) Tel. +44(0)2920 876758

**S7 Fig: Change from vehicle following treatment with risperidone or haloperidol.**

| 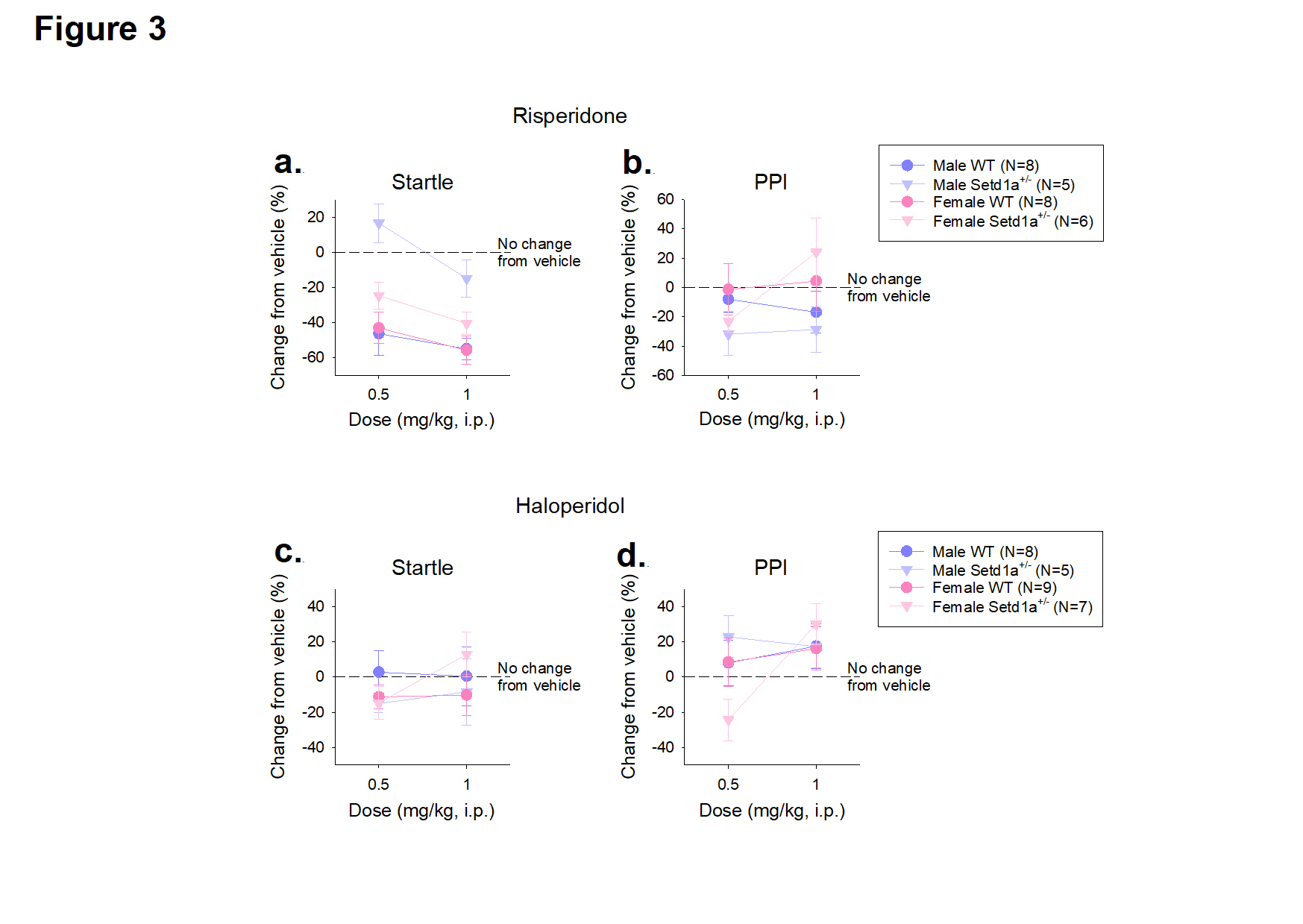 |
| --- |
| The effects of risperidone (a, b) and haloperidol (c, d) on startle responding and prepulse inhibition (PPI) were further demonstrated by calculating the proportional change of each dose of drug relative to vehicle administration. For risperidone, a significant main effect of DOSE (F_1,23_=9.638, p=0.005, µ^2^=0.295) indicated that the 1 mg/kg dose attenuated startle responding (a) more than the 0.5 mg/kg dose. Within this effect, a significant GENOTYPE*SEX interaction (F_1,23_=5.177, p=0.033, µ^2^=0.184), highlighted a different pattern of startle responding in male *Setd1a*^+/-^ mice relative to female *Setd1a*^+/-^ mice and both sexes of WT subjects. Individual paired means analysis vs. no change (0%) indicated significant decreases in startle responding at both doses of risperidone in female *Setd1a*^+/-^ mice (t_5_=3.31, p=0.021 and t_5_=6.44, p=0.001, for 0.5 mg/kg and 1 mg/kg, respectively) and male (t_7_=3.72, p=0.008, and t_7_=8.91, p=0.001) and female (t_7_=4.85, p=0.002 and t_7_=6.88, p=0.001) WT mice. Although male *Setd1a*^+/-^ mice demonstrated an increase from vehicle at 0.5 mg/kg, and a small decrease in startle responding at 1 mg/kg, neither of these effects were significant from “no change from vehicle” (p>0.05). These data further demonstrated that risperidone treatment an either dose used was without significant effect of PPI (b, main effect of DOSE, F_1,23_=2.286, p=0.144, µ^2^=0.090), a similar effect in both WT and *Setd1a*^+/-^ mice (main effect of GENOTYPE, F_1,23_=0.411, p=0.528, µ^2^=0.018). As reported in the main text, haloperidol was without significant effect on startle responding (a, main effect of DOSE, F_1,25_=2.45, p=0.13, µ^2^=0.09) or PPI (b, main effect of DOSE, F_1,25_=4.06, p=0.06, µ^2^=0.14). There were also no differences between WT and *Setd1a*^+/-^ mice (main effect of GENOTYPE, F_1,25_=0.02, p=0.881, µ^2^=0.001 and F_1,25_=0.01, p=0.929, µ^2^=0.001, for startle and PPI, respectively). There were no significant difference from “no change from vehicle” for either dose of haloperidol on startle responding or PPI in both male and female WT and *Setd1a*^+/-^ mice. For the drug studies, responses in the 8 and 16 dB prepulse trials were combined to generate a single value for ease of analysis and Cohort 2 was divided into separate groups for each drug study. * shows significant main effect of GENOTYPE at p<0.05. Data shows mean±SEM. |

**End of document**
