# Supplementary material for: Sex-dependent effects of Setd1a haploinsufficiency on development and adult behaviour
Source: PLoS One. 2024 Aug 14;19(8):e0298717. doi: 10.1371/journal.pone.0298717 (PMC11324134; doi:10.1371/journal.pone.0298717)
Supplement: S1 Table — (DOCX) [file pone.0298717.s008.docx]

**Sex-dependent effects of *Setd1a* haploinsufficiency on development and adult behaviour**

Matthew L. Bosworth^1^, Anthony R. Isles^1^, Lawrence S. Wilkinson^1,2,3^, & Trevor Humby^1,2,3^*

^1^MRC Centre for Neuropsychiatric Genetics and Genomics, Division of Psychological Medicine and Clinical Neuroscience, School of Medicine, Cardiff University, Cardiff, UK

^2^School of Psychology, Cardiff University, Cardiff, UK

^3^Neuroscience and Mental Health Research Institute, Cardiff University, Cardiff UK

*Corresponding author: Dr Trevor Humby [HumbyT@cardiff.ac.uk](mailto:HumbyT@cardiff.ac.uk) Tel. +44(0)2920 876758

**S1 Table: Samples sizes for evaluation of embryo and placenta size**

|  | **Number of litters** | **WT Male** | ***Setd1a*^+/-^ Male** | **WT Female** | ***Setd1a*^+/-^ Female** | **Total** |
| --- | --- | --- | --- | --- | --- | --- |
| **E11.5** | 4 | 11 | 5 | 6 | 6 | 28 |
| **E13.5** | 5 | 13 | 11 | 14 | 8 | 46 |
| **E18.5** | 6 | 10 | 11 | 10 | 13 | 44 |

**End of document**
