# Supplementary material for: Sex-dependent effects of Setd1a haploinsufficiency on development and adult behaviour
Source: PLoS One. 2024 Aug 14;19(8):e0298717. doi: 10.1371/journal.pone.0298717 (PMC11324134; doi:10.1371/journal.pone.0298717)
Supplement: S2 Table — (DOCX) [file pone.0298717.s009.docx]

**Sex-dependent effects of *Setd1a* haploinsufficiency on development and adult behaviour**

Matthew L. Bosworth^1^, Anthony R. Isles^1^, Lawrence S. Wilkinson^1,2,3^, & Trevor Humby^1,2,3^*

^1^MRC Centre for Neuropsychiatric Genetics and Genomics, Division of Psychological Medicine and Clinical Neuroscience, School of Medicine, Cardiff University, Cardiff, UK

^2^School of Psychology, Cardiff University, Cardiff, UK

^3^Neuroscience and Mental Health Research Institute, Cardiff University, Cardiff UK

*Corresponding author: Dr Trevor Humby [HumbyT@cardiff.ac.uk](mailto:HumbyT@cardiff.ac.uk) Tel. +44(0)2920 876758

**S2 Table: Details of the litters, genotype and sex ratios of the samples used in the pre- and post-natal assessments.**

|  | **Number of litters** | **Median**  **litter size** | **WT:Setd1a^+/-^** | | **Males:Females** | |
| --- | --- | --- | --- | --- | --- | --- |
|  |  |  | **(Mean±SEM)** | **Chi^2^ test** | **(Mean±SEM)** | **Chi^2^ test** |
| E11.5 | 4 | 7 | 0.39±0.09 | *X^2^_2_*=0.50, p>0.05 | 0.57±0.05 | *X^2^_3_*=0.00, p>0.05 |
| E13.5 | 5 | 10 | 0.43±0.08 | *X^2^_3_*=0.00, p>0.05 | 0.53±0.03 | *X^2^*_4_=0.60, p>0.05 |
| E18.5 | 6 | 7 | 0.55±0.07 | *X^2^*_4_=0.67, p>0.05 | 0.49±0.12 | *X^2^*_5_=0.00, p>0.05 |
| Postnatal (P28) | 13 | 7 | 0.45±0.07 | *X^2^*_10_=1.39, p>0.05 | 0.44±0.07 | *X^2^*_10_=1.39, p>0.05 |

Note: WT: Setd1a^+/-^ ratio is the of number of Setd1a^+/-^ samples/total samples and M:F ratio is calculated as number of male samples/total samples.

**End of document**
