# Supplementary material for: Sex-dependent effects of Setd1a haploinsufficiency on development and adult behaviour
Source: PLoS One. 2024 Aug 14;19(8):e0298717. doi: 10.1371/journal.pone.0298717 (PMC11324134; doi:10.1371/journal.pone.0298717)
Supplement: S3 Table — (DOCX) [file pone.0298717.s010.docx]

**Sex-dependent effects of *Setd1a* haploinsufficiency on development and adult behaviour**

Matthew L. Bosworth^1^, Anthony R. Isles^1^, Lawrence S. Wilkinson^1,2,3^, & Trevor Humby^1,2,3^*

^1^MRC Centre for Neuropsychiatric Genetics and Genomics, Division of Psychological Medicine and Clinical Neuroscience, School of Medicine, Cardiff University, Cardiff, UK

^2^School of Psychology, Cardiff University, Cardiff, UK

^3^Neuroscience and Mental Health Research Institute, Cardiff University, Cardiff UK

*Corresponding author: Dr Trevor Humby [HumbyT@cardiff.ac.uk](mailto:HumbyT@cardiff.ac.uk) Tel. +44(0)2920 876758

**S3 Table: Supporting data for the novel object recognition test.**

| **30 mins** | | **24 hours** | |  |
| --- | --- | --- | --- | --- |
| **Dependent variable** | **WT** | **KO** | **WT** | **KO** |
| Acquisition time (mins) | 6.9 (3.8) | 7.3 (4.2) | 7.1 (4.0) | 6.9 (4.1) |
| Novel object exploration time (s) | 19.2 (9.0) | 20.1 (11.0) | 8.1 (5.2) | 8.0 (5.4) |
| Familiar object exploration time (s) | 10.9 (8.7) | 9.5 (6.8) | 5.7 (4.0) | 6.1 (3.6) |

Note: Mean (SD) of acquisition time (time taken to achieve 40 seconds of object exploration) and the individual object exploration times at test for the 30 minute and 24 hour retention intervals.

**End of document**
